# Lumbar Spinal Angiolipoma with Expanding Left Neural Foramen Mimicking Lumbar Schwannoma; Case Report and Review of The Literature

**DOI:** 10.2174/1874205X01711010020

**Published:** 2017-09-26

**Authors:** Yener Akyuva, Aylin Gonultas, Numan Karaaslan, Zehra Gulciftci Dagci, Semih Saglik, Mehmet Isyar, Mahir Mahirogullari

**Affiliations:** 1 Republic of Turkey, Ministry of Health, State Hospital, Department of Neurosurgery, 59100, Tekirdag, Turkey; 2 Republic of Turkey, Ministry of Health, State Hospital, Department of Pathology, 59100, Tekirdag, Turkey; 3 Namik Kemal University School of Medicine, Department of Neurosurgery, 59030, Tekirdag, Turkey; 4 Republic of Turkey, Ministry of Health, State Hospital, Department of Radiology, 56100, Siirt, Turkey; 5 Medicalpark Bahcelievler, Department of Orthopaedic and Traumatology, 34050, Istanbul, Turkey; 6 Istanbul Memorial Health Group, Department of Orthopaedic and Traumatology, 34758, Istanbul, Turkey

**Keywords:** Lumbar angiolipoma, Schwannoma, Spinal angiolipoma, Spinal cord, Spinal canal

## Abstract

**Aim:**

To describe a patient with lumbar angiolipoma mimicking schwannoma in the posterolateral side of the spinal canal with expansion of the left lumbar foramen and to discuss the clinical, radiologic, and surgical features of these lesions with literature.

**Methods:**

Without language restriction in this paper, the electronic databases; The Cochrane Collaboration the Cochrane, The Cochrane Library (Issue 2 of 12, Feb. 2011), ProQuest, US National Library of Medicine, National Institutes of Health (NLM) and PubMed dating from 1966 September to January Week 2 2017, were searched for comparative experimental studies using the terms: “OR”, “AND”. On-line literature searches were conducted using the key words “lumbar angiolipoma”, “schwannoma “, “spinal angiolipoma”, “spinal cord”, and “spinal canal”. We compared this research with our patient.

**Results:**

Bilateral L2 total laminectomy, excision of the tumors and bilateral L2-L3 transpedicular stabilization were performed, and complaints improved prominently. Pathological examination was reported as angiolipoma.

**Conclusion:**

The research shows that a probable diagnosis in such tumor cases could be made by sufficient pre-op scanning before surgical operations and although angiolipoma has been rarely seen in lumbar posterolateral space, it can be seen in lumbar region and mimic schwannoma as producing symptoms and signs of spinal cord and nerve root compression.

## 1 INTRODUCTION

Angiolipoma is a frequent benign lipomatosis tumor comprising of mature fatty tissue and vascular proliferation. They generally occur under the skin. 2/3 of the cases are forearm based. Besides, they locate in the body and arm [1]. Angiolipoma is rarely seen in the spinal canal [2, 3].

Spinal angiolipoma is a rare specific clinicopathologic entity, which is to be distinguished from skin angiolipoma [3]. Spinal angiolipomas comprises 0.04%-1.2% of all spinal cord tumors and spinal 2-3% of extradural tumors [2, 4-7]. Most of spinal angiolipomas are seen in thoracic region of posterior extradural areas [5, 8]. Lumbar spinal angiolipomas are rarely seen and comprise 9.6% of all spinal angiolipomas [5, 9, 10]. Besides, lumbar spinal angiolipomas are frequently seen in anterior extradural locations [9].

Spinal angiolipomas do not often show infiltration, yet in some cases, infiltration to soft surrounding tissues and bone may occur [9, 11]. Since angiolipoma is rarely seen in the spinal canal, it may not be noticed in the evaluation of lesions in this area [8]. It may sometimes mimic a malign tumor due to its infiltrative appearance in soft surrounding tissues and vertebra [8, 12]. Also, hydatid cyst, neurofibroma and hemangioma should be kept in mind as a differential diagnosis [13-15].

Angiolipoma with lumbar posterolateral location is an extremely rare tumor [8, 11]. In posterolateral location of lumbar region at spinal canal most frequently encountered primary tumor is schwannoma [16-18]. However, when the full texts of these researches are analyzed, it is not found that, as in our study, angiolipoma mimics lumbar schwannoma due to surrounding lumbar root and expanding neural foramen.

Schwannoma, which is known as norilemma, is based on benign peripheral nerve sheath stemming from a schwann cell. This is the most common solitary tumor of the peripheral nerves [16, 18]. Malignant degeneration of the schwannoma is very rare, yet there are few reports in which malign transformation is mentioned. Schwannoma, which can be seen in every area of extradural peripheral nerves, is frequently seen in the nerve root in the extradural space [10, 18].

The present study aimed to analyze the patient that mimics lumbar schwannoma, which is extradural posterolateral located in the lumbar area before the operation. In addition, it was intended to indicate spinal angiolipoma case, which arose suspicion regarding its malignity, along with a literature review as they infiltrated to adjacent bone and soft tissues which were seen during the operation.

## 2 CASE REPORT

The patient was a 65-year-old-woman with body mass index 23 kg/m^2^. The history of smoking and alcohol did not present in the patient. The patient was referred to our clinics with back pain and having difficulty in walking due to weakness in her left leg which had aggravated last 3 months. After careful anamnesis, it was found that the patient had a history of the left hemiparesis that had started one year ago, due to the right-sided thalamic cerebrovascular occlusion. However, during the neurological examination, it was discovered that lower extremity weakness was relatively more significant than that was seen in the upper extremity.

While nearly normal muscle strength was found due to cerebrovascular occlusion in the left upper extremity, 3/5 muscular force was detected in the left lower extremity of proximal extensor muscle groups. The patient had difficulty of knee extension which suggested primarily a pathology of L3 nerve root. Following examination of positive leg test, it was discovered that there was a motor deficit in lower extremity.

In laboratory tests, no result was observed to consider hematologic malignity. However, it was not excluded the fact that the pathologic diagnosis was crucial for lymphoma. The biological and hormone values were found to be in the limits.

A Magnetic Resonance Image (MRI) scan of the patient was immediately taken and a tumor was seen in L2-L3 disc level. It was in the left posterolateral extradural area and its long axis was parallel to the spinal cord.

**Fig. (1):**
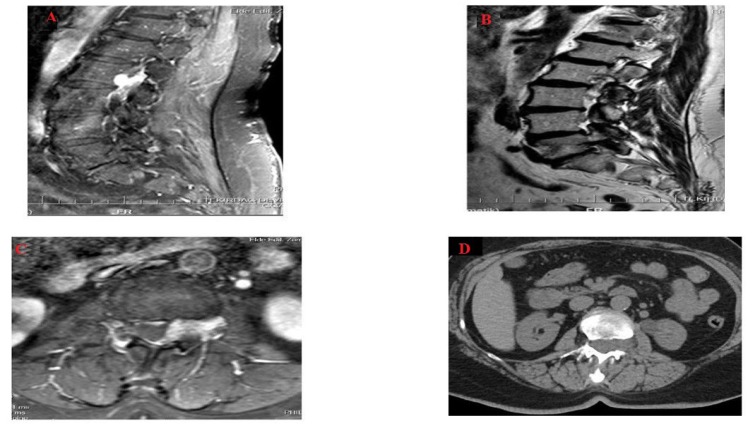
Radiological findings of patient with left L2-3 postero-lateral angiolipoma.

The tumor diameter was 3.2 cm in craniocaudal plane and it shrank on both sides. Anteroposterior diameter was 0.8 cm on the largest zone and the dural sac was found to be compressed slightly and postero-laterally by this mass (Figs. **1A**-**1C**). Moreover, there were heterogeneous hyperintensity in T1 and T2 weighted images detected on MRI, which expanded along left L2-L3 neural foramen causing extension in neural foramen whereas there were hypo intense focuses on T2-weighted images (Figs. **1A** and **1C**). Following contrast agent administration, in T1-weighted fat suppressed images, it was found that there was a lesion in which there was an intensive contrast enhancement.

The lumbar computed tomography (CT) of the patient was also performed, it was found that lesion was seen isodense with spinal cord and caused extension in the left L2-L3 neural foramen and stenosis of the spinal canal (Fig. **1D**). The patient was diagnosed with the tumor, which was thought to be schwannoma or lymphoma before the operation. Tumor excision was then performed and stabilization was ensured by using bilateral L2 total laminectomy, left L2/L3 facetectomy technique and bilateral L2-L3 transpedicular screw rod system [18, 19].

The material excised after the operation was transferred to the laboratory in a container with 10% formol solution to realize histopathological examination. It was found in the macroscopic examination that the materials were comprised of five yellow-pink bleeding tissues sized from 0.8 to 0.2 cm. The full extent of the material was sampled. The tissue pieces were embedded in paraffin blocks after follow-up. Five micrometer sections were taken from paraffin-embedded tissues, stained using the Hematoxylin and Eosin (H&E) and examined under the light microscope.

**Fig. (2):**
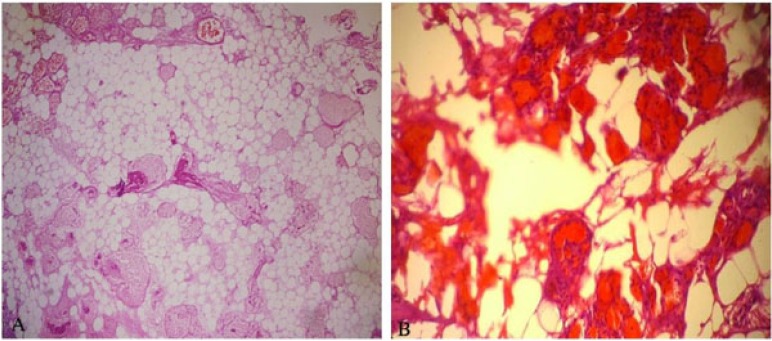
***A;*** Fibrine thrombus inside vascular component and mature lipoid tissue (Magnification; x4, H&E). ***B;*** Mature lipoid tissue between vascular component with congestion (Magnification; x20, H&E).

Cautery artefact was seen in tissue fragments during histological examination. Besides, mature adipose tissue and tumor composed of abnormal vascular proliferation were mostly seen in the capillary structure. Fibrin thrombus was also the case in lumens of some capillary vessels. There wasn’t atypia and mitosis but abundant adipose tissue was seen in component of resected tumor tissue (Figs. **2A** and **2B**).

In immunohistochemically examination, cluster of differentiation (CD)-34 and S100 were detected as well as positive stain. Stain with LCA and pancytokeratin were not detected. The Ki-67 proliferation index was found low.

The patient was evaluated in line with angiolipoma. There weren’t infiltration of extradural soft tissues and bone.

## 3 DISCUSSION WITH LITERATURE REVIEW

The first spinal angiolipoma case was reported in 1890 by Berenbruch [3, 28]. Howard and Helwig defined angiolipoma as a clinicopathologic entity including vascular and mature adipose elements in 1960 [2, 4].

Lin *et al.* divided spinal angiolipoma into two groups: noninfiltrative and infiltrative [4, 7]. The noninfiltrating spinal angiolipomas are frequently encapsulated and occur mostly in the posterior extradural space of the spinal cord. The infiltrating types are rarely seen and partly or completely unencapsulated. They are located in the anterior and anterolateral extradural space of the spinal cord [7]. The prognosis of the noninfiltrating spinal angiolipomas is better than infiltrating types and they have an unfavorable prognosis [2, 4].

The histogenesis of angiolipomas are not known. These tumors are most likely to be caused by abnormal primitive pluripotential mesenchymal cells which are differentiating in lipomatosis, angiomatous and mix tissues [2]. Tumor is macroscopically encapsulated or unencapsulated red soft tissue in pathologic evaluation [20].

In histological incisions, mature adipocytes and vessel proliferation comprised of vessels at the size of branching capillary are seen [1]. In some parts of vessels, there are fibrin thrombus as diagnostic characteristic. There are distinctive mast cells in angiolipomas. Fibrin thrombus and mast cells are morphologic findings, which is a distinctive characteristic from lipoma [3].

In tumor, the rate of fat tissue to the vascular compartment ranges between 1/3 and 2/3 [7, 8, 21]. Vascular structures in some angiolipomas comprise almost the whole of the tumor. These tumors that are called ‘cellular angiolipoma’ are to be separated from kaposi sarcoma and angiosarcoma [1]. It is different from these tumors because lipoid tissue, pleomorphism, atypia and mitosis are not seen in angiolipoma [6, 20].

Immunohistochemical examination from the literature was performed in some cases and Ki-67 index is also lower in angiolipoma [6, 20, 22]. Spinal angiolipomas are generally seen between the ages of 40 and 60 and especially in women [6, 7]. There are few cases seen in children [2].

Almost all spinal angiolipomas are localized in extradural site. Most of these extradural angiolipomas are in posterior part in thoracic site [11]. Thoracolumbar component and pure lumbar localization is rare [22]. Tumors in lumbar area generally locate in anterior of spinal canal [11]. Today MRI is the most appropriate device in diagnosing angiolipomas. In MRI angiolipomas are mostly seen heterogenous hypo intense in T1-weighed examination on extradural area. In T2-weighted examination, fatty components are seen as hyper intense and vascular areas as hypo intense. More specifically, after contrast agents such as gadolinium are administered, lipoid layer and vascular flow openings are seen, which become distinctive in T1-weighted images [23, 24]. Schwannoma is seen heterogeneously hyperintense in T2-weighted images and intense contrast enhancement in T1 weighted images [16, 19].

In literature, it is reported that typical angiolipoma is radiolucent in both plain radiography and CT. In addition, CT is adjuvant imaging method since vascular enclosing radiolucent may contain calcific sites [25, 26]. Also, schwannoma may have calcific foci on CT and intense contrast enhancement is usually seen [16, 18].

MRI and CT devices were used in order to diagnose the patient in the present study. Similar images that had been defined were seen in MRI images that were carried out by contrasted pharmacologic agents (Figs. **1A** and **1D**). Also, CT show isodense mass with calcific foci expanding left neural foramen. L2 vertebrae foraminal enlargement could be understood on plain film. Multi-modality imaging should be performed on spinal epidural angiolipoma as well as on the breast [17].

Patient of this case had no family history, skin neurofibroma, 'cafe-au-lait spots' skin lesions, Lisch Nodule or optic glioma, but neurofibroma had not excluded with a low differential diagnosis. Because neurofibroma and as well as schwannoma may have similar radiological appearance on CT and MRI.

Angiolipomas cannot be easily excised like lipoma because they are slim with irregular capsule. Wide local excision of angiolipoma minimizes the risk of relapse. Wide resection may require excision of a significant part of healthy tissue containing critical neurovascular structures. Excision of an important nerve or vessel is to be operated by considering high incidence of local recurrence of the tumor that was insufficiently excised with the purpose of gain wider bounds. For this reason, the surgery should be individualized [27].

Application of tumor excision by laminoplasty and/or laminectomy in the area of tumor is a standard treatment modality in extradural located angiolipoma operations [28, 29].

However, in literature, in the surgery of tumors with extraforaminal component, it is emphasized that facetectomy might be applied in treatment of tumors for the extraforaminal component. Afterwards, stabilization is recommended using transpedicular screw in order to prevent lumbar instability after tumor excision [19, 30]. Unlike the cases in the literature who were operated the patient in the present study was performed excision as the secondary technique since operated tumor surrounded lumbar root and had extraforaminal component by expanding neural foramen, thus imitating lumbar schwannoma.

Also, the pre-operative malign tumor was suspected because of invasive radiological appearance, marginal resection of the tumor was planned and carried out in this patient. So that posterior transpedicular stabilization should had done.

In literature, there are some studies indicating that malign transformation of angiolipomas may rarely seen [27]. Although preoperative radiological findings consisted with malignancy, it was reported that there wasn’t malignity in the excised tumor tissue. Yamashita *et al.* studied characterization of histological types in order to contribute to treatment modalities in spinal cord tumors before operations [31]. Similarly, pre-op MRI was used when diagnosing the patient in our study. Besides, postop excised tissue material was evaluated histopathologically.

## CONCLUSION

As a result, it was found that a probable diagnosis in such tumor cases could be made by sufficient pre-op scannings before surgical operations and total excision and full treatment could be sustained by means of correct surgical techniques. Although angiolipoma has been rarely seen in lumbar posterolateral space, it can be seen in lumbar region and mimic schwannoma as producing symptoms and signs of spinal cord and nerve root compression.
